# 

**Published:** 1903-02

**Authors:** 


### BOOKS RECEIVED.

Book on the Physician Himself, and Things that Concern His Reputation and Success. By D. W. Cathell, M. D. The Twentieth Century Edition. Eleventh Edition, revised and enlarged by the author and his son, William T. Cathell, A. M., M. D. Pp. 411, royal octavo. Philadelphia: F. A. Davis Company. 1903. [Cloth, $2.50 net, delivered ]

Surgical Anatomy and Operative Surgery. For Students and Practitioners. By John J. McGrath, M. D., Professor of Surgical Anatomy and Operative Surgery at the New York Post-Graduate Medical School; Visiting Surgeon to the Harlem Hospital, and Assistant Visiting Surgeon to the Columbus Hospital, New York. Illustrated with 227 illustrations, including colors and half-tones. Pages xiv-359. Royal Octavo. Philadelphia: F. A. Davis Company. 1903. [Extra cloth, $4.00 net; sheep, or half-Russia, $5.00 net, delivered.]

Culbreth's Materia Medica and Pharmacology. Third edition. A Manual of Materia Medica and Pharmacology. Comprising all Organic and Inorganic Drugs, which are and have been official in the United States Pharmacopeia, together with important Allied Species and Useful Synthetics. By David M. R. Culbreth, M. D., Professor of Botany, Materia Medica, and Pharmacognosy in the Maryland College of Pharmacy, Professor of Materia Medica and Pharmacognosy in the University of Maryland Medical and Dental Schools, Baltimore. Third edition enlarged and thoroughly revised. In one octavo volume of 905 pages, with 473 illustrations. Philadelphia and New York: Lea Brothers & Co. 1903. [Cloth, $4.75 net.]

Therapeutics of Dry Hot Air. By Clarence Edward Skinner, M. D., LL. D., New Haven, Conn., Professor of Thermotherapy in the New York School of Physical Therapeutics; Editor of the Department of Thermotherapy in the Journal of Advanced Therapeutics; Physician in charge of the Newhope Hot Air Sanitarium, New Haven Conn. Octavo, pp. 200. Illustrated. New York: A. L. Chatterton & Co. 1903. [Price, $2.00.]

Lea's Series of Pocket Textbooks. A Pocket Textbook of Anatomy. By William H. Rockwell, Jr., M. D., Assistant Demonstrator of Anatomy, College of Physicians, Columbia University, New York. In one 12mo volume of 600 pages, with 70 illustrations. Edited by Bern B. Gallaudet, M. D. Philadelphia and New York: Lea Brothers & Co. 1903 [Price, cloth, $2.25 net; limp leather, $2.75 net.]

Atlas and Epitome of Diseases of the Mouth, Pharynx and Nose. By Dr. L. Grunwald, of Munich. From the second revised and enlarged German edition. Edited, with additions, by James E. Newcomb, M. D., Instructor in Laryngology, Cornell University Medical School; Attending Laryngologist to the Roosevelt Hospital, Out-Patient Department. With 102 illustrations on 42 colored lithographic plates, 41 text-cuts and 219 pages of text. Philadelphia and London: W. B. Saunders & Co. 1903. [Cloth, $3.00 net.]

Atlas and Epitome of Human Histology and Microscopic Anatomy. By Privatdocent Dr. J Sobotta, of Wurzburg. Edited, with additions, by G. Carl Huber, M. D., Junior Professor of Anatomy and Histology, and Director of the Histological Laboratory, University of Michigan, Ann Arbor. With 214 colored figures on 80 plates, 68 text-illustrations, and 248 pages of text. Philadelphia and London: W. B. Saunders & Co. 1903. [Cloth, $4.50 net.]

Biographic Clinics. The Origin of the Ill Health of DeQuincey, Carlyle, Darwin, Huxley and Browning. By George M. Gould, M. D., Editor of American Medicine; Author of "An Illustrated Dictionary of Medicine." Duodecimo, pp. 223. Philadelphia: P. Blakiston's Son & Co. 1903. [Price, $1.00.]

The Practical Medicine Series of Year Books. Comprising ten volumes on the year's progress in medicine and surgery. Issued monthly under the general editorial charge of Gustavus P. Head, M. D., Professor of Laryngology and Rhinology in the Chicago Post-Graduate Medical School. Volume III. The Eye, Ear, Nose and Throat. Edited by Casey A. Wood, C. M., M. D., Albert H. Andrews, M. D., T. Melville Hardie, A. M., M. D. Chicago: The Year Book Publishers. 1902. [Price, $1.50; entire series, $7.50.]

The Johns Hopkins Hospital Reports. Volume X. Nos. 6, 7, 8, 9. Baltimore: The Johns Hopkins Press. 1902.

The Practical Medicine Series of Year Books. Comprising ten volumes on the year's progress in medicine and surgery. Issued monthly under the general editorial charge of Gustavus P. Head, M. D., Professor of Laryngology and Rhinology in the Chicago Post-Graduate Medical School. Volume II. General Surgery. Edited by John B. Murphy, M. D., Professor of Surgery, Northwestern University Medical School Chicago: The Year Book Publishers. 1902. [Price, $2.00; entire series, $7.50.]

Transactions of the Twenty-Fourth Annual Meeting of the American Laryngological Association, held at Boston, Mass , May 26, 27 and 28, 1902. James E. Newcomb, M. D., Secretary. New York: Rooney & Otten Printing Co. 1902.

Transactions of the American Surgical Association. Twentieth annual meeting, held at Albany, June 3-5, 1902. Richard H. Harte, M. D., Recorder of the Association. Philadelphia: William J. Dornan, Printer. 1902.

Report of the Commissioner of Education for the Year 1900-1901. Volume I. Washington: Government Printing Office. 1902.

